# Unusual Bilateral Oblique Craniofacial Cleft with Atypical Cleft of Palate: A Case Report with 3 Years of Follow-Up

**DOI:** 10.1055/s-0045-1811204

**Published:** 2025-09-22

**Authors:** Bharatendu Swain, Shalini Sampreethi, Srujana Gorle, Tajaly Saneen

**Affiliations:** 1 Department of Plastic and Reconstructive Surgery, Aakar Asha Hospital, Hyderabad, Telangana, India

**Keywords:** atypical cleft palate, oblique craniofacial cleft, macrostomia

## Abstract

Oblique facial clefts are a rare entity and can be associated with palatal anomalies like synechiae caused by amniotic bands. Their treatment is individualized following sound reconstructive surgical principles. This is a case report of a 15-month-old child with cleft palate in which the right palatal shelf was represented by a tongue-like projection, associated with macrostomia and bilateral medial and lateral oro-ocular clefts. The palatal cleft was closed in two stages and the oblique clefts in the first stage. At 4 years of age, the velopharyngeal incompetence was addressed by a superiorly based pharyngeal flap. The macrostomia was corrected by a sliding V-Y flap, which effectively lengthened the buccal mucosa and ensured oral competence.

## Introduction

Craniofacial clefts are disfiguring developmental anomalies of tissue excess, tissue deficiency, or even normal but separated tissues along the lines of face and cranium. 1 2 3 4 Many attempts have been made in the past by American Association of Cleft Palate Rehabilitation, Boo-Chai, Karík, and van der Meulen et al to classify this heterogeneous cleft malformation. Tessier's classification, following well-defined zones of face and orbit along an embryological map, has proven to be most complete and withstood the test of time. 1 5 6 7 8 Tessier considered eyelid (palpebral opening) and orbits as the landmark to divide the face into upper and lower hemispheres. He thus numbered 0–14 clefts as 0–7 for south-bound/lower hemisphere or facial clefts and 8–14 as north-bound/upper hemisphere or cranial clefts. These clefts are often noted in combination of 0–14, 1–13, 2–12, 3–11 ,4–10, 5–9, and 6–8. Soft tissue clefts may be accompanied by bony clefts, but are seldom affected to the same degree.

Tang et al suggested an interesting spectacle frame diagram that factors the bony involvement as an addition to Tessier's classification. 9

Oblique facial clefts are reported to comprise 0.25% of all facial clefts and can be classified into three main types: naso-ocular, medial oro-ocular, and lateral oro-ocular. 10 Among the oblique facial clefts, Tessier no. 5 is the rarest. 11

## Case Report

**Fig. 1:**
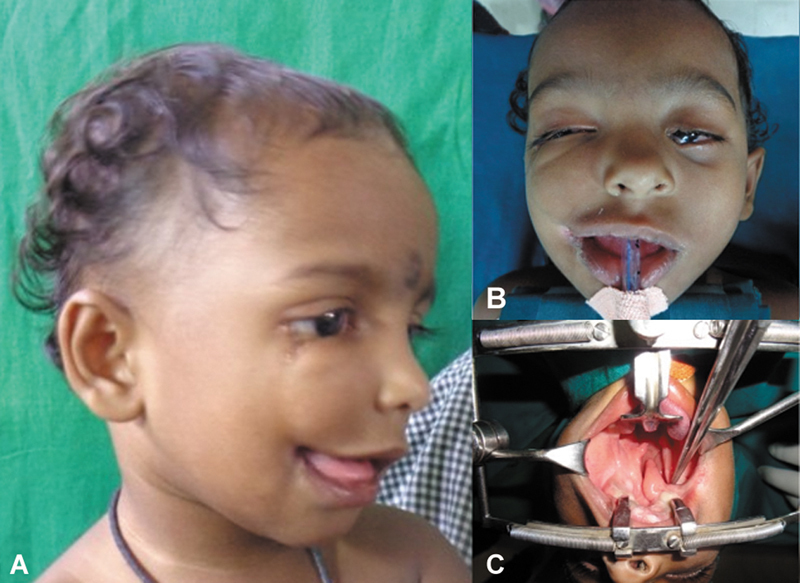
**(A)** Cleft extending from near right angle of mouth to right lateral lower eyelid with macrostomia. **(B)** Intra-op picture showing macrostomia and coloboma of left upper and lower eyelids. **(C)** Cleft of palate with free-lying, uvula-like projection within the cleft gap, extending to the buccal mucosa behind the right upper second premolar with loss of sulcus at the site of attachment.

A 15-month-old full-term female baby, first born of healthy nonconsanguineous parents, presented in April 2022 with facial anomalies. There was neither history of facial abnormalities in the family nor implicated teratogenic factors. There was asymmetrical bilateral oblique facial cleft with abnormal cleft of palate. On the right side, there was macro-stomia and the cleft commenced from near the angle of the mouth, causing subsurface meloschisis and terminated as a cleft of the lateral third of the lower eyelid ( Fig. 1A ). On the left side, there was coloboma of left lower eyelid extending between the medial canthus and inferior lacrimal punctum and of the left upper eyelid involving the medial third ( Fig. 1B ). Intra-oral examination revealed complete cleft of hard and soft palate with an atypical tongue-like soft tissue projection in the cleft gap; it was hanging in the midline dividing the major cleft, its base continuing as a bowstringing fibro-mucosal band splitting the alveolar ridge behind the right upper cuspid and continuing to the right oral commissure obliterating the gingivobuccal sulcus; there was no continuity with the nasal septum ( Fig. 1C ).

## Intervention

**Fig. 2:**
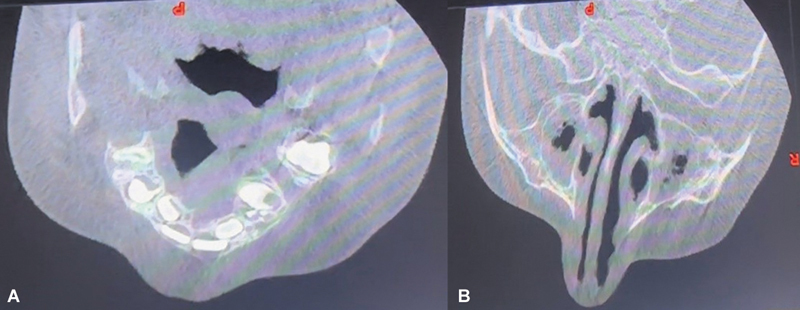
**(A)** CBCT maxilla showing cleft between right upper second premolar and first molar teeth. **(B)** CBCT maxilla showing bony hypertrophy of zygoma on the right side. CBCT, cone beam computed tomography.

Cone-beam computed tomography scan of the maxilla was advised when the child presented at 4 years of age. 12 A cleft was noted in the alveolus between the right upper cuspid and first molar teeth, not extending into the maxillary sinus ( Fig. 2A ). At the upper part of right zygoma and inferior orbital rim, bony hypertrophy was noted ( Fig. 2B ). There were no associated limb or cardiac/visceral anomalies.

**Fig. 3:**
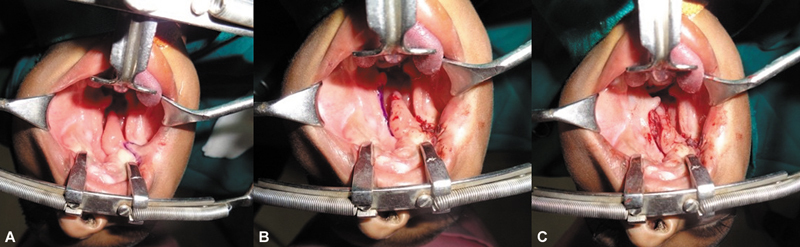
**(A)** Attachment of tongue-like projection to the buccal mucosa with loss of sulcus at the site of attachment. **(B)** Release of tongue-like projection from the buccal mucosa near right lateral commissure and creation of sulcus. **(C)** Suturing of left cleft margin to the tongue-like projection by raising proper muco-periosteal flaps from left cleft palate in stage 1.

**Fig. 4:**
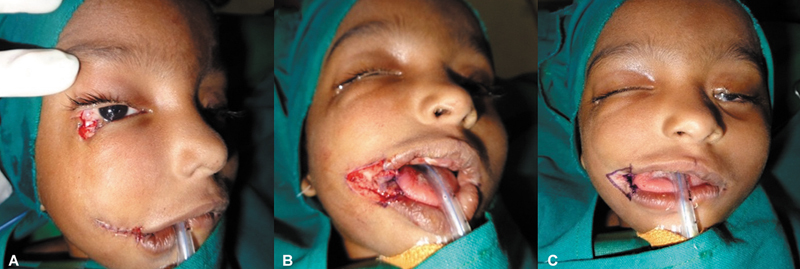
**(A)** Right commissuroplasty markings. **(B)** V-Y incision and advancement to correct macrostomia and restore buccal mucosa length. **(C)** Repair of right lower eyelid coloboma by diamond-shaped incision.

**Fig. 5:**
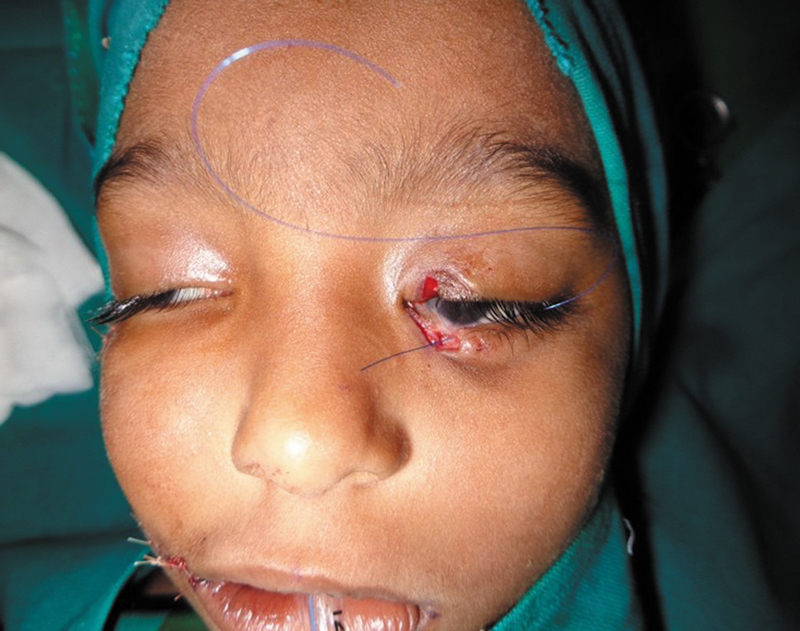
Left lower eye lid punctum threaded before duct anastomosis and coloboma closure.

As the child presented at 15 months age, treatment of cleft palate was a priority. The left margin of the tongue-like projection was merged with the major palatal shelf cleft margin; muco-periosteal dissection and layered closure were done. The palate gap on the right side was left alone ( Fig. 3A – C ). In the same sitting, the area of skin enclosed between the points for macrostomia closure was incised and advanced into the mouth in a V to Y fashion to elongate the tight mucosal band ( Fig. 4A ). The repair of coloboma of both right lower eyelid and left upper eyelid was done by using diamond-shaped incisions ( Fig. 4B , C ). A shield incision was made on left lower eyelid, inferior canicular punctum was cannulated, and ends of the lacrimal duct anastomosed with Ethilon 8–0 suture under operating microscope ( Fig. 5 ).

**Fig. 6:**
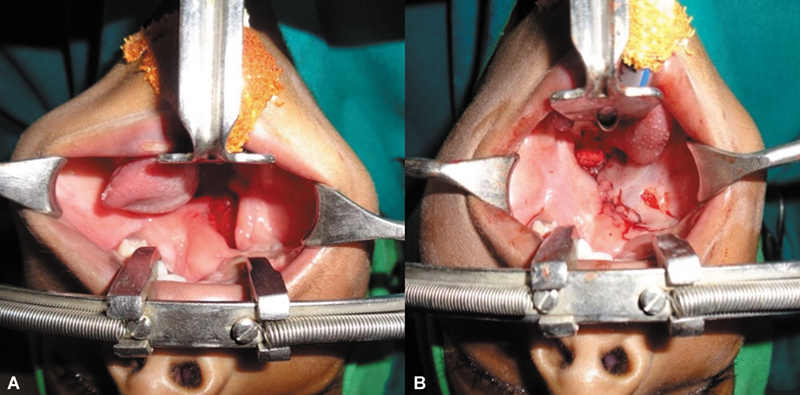
**(A)** Stage 2 intra-op picture of fused left cleft palate shelf with the tongue-like projection. **(B)** Closure of right cleft palate after 6 months.

Six months later, the tongue-like palatal projection on the right side was merged with the previously repaired palate in layers ( Fig. 6A , B ).

## Follow-Up and Further Intervention

**Fig. 7:**
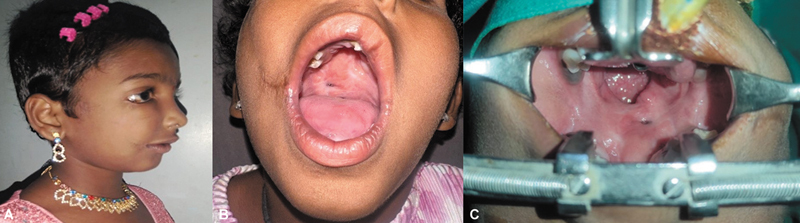
**(A)** Two-year follow-up. Improved appearance on the right side. **(B)** Normal mouth opening with commissural function. **(C)** Small palatal fistula and larger velopharyngeal aperture on the left side.

On review 3 years later, the macrostomia and colobomas had improved in appearance with good oral competence ( Fig. 7A ). The right subsurface facial cleft was less grooved. Pterygium was noted in both eyes, more severe on the right, with a band extending to repaired coloboma site ( Fig. 7B ). The inferior orbital rim and adjoining malar bone on the right side were prominent. There was a small posterior palatal fistula and a larger velopharyngeal gap on the left side ( Fig. 7C ). Articulation was moderate, with hypernasality.

The palatal fistula was closed in two layers and velo-pharyngeal incompetence addressed with superiorly based pharyngeal flap attached to the posterior edge of the soft palate integrating its muscle with levator muscle. The pterygium of the right eye was excised; protruding inferior orbital rim and upper zygoma chiseled to attain symmetry. The groove near the right oral commissure was addressed by subcision and dermis-fat grafting.

## Discussion

Several types of oblique clefts have been reported by many authors. 6 13 14 The etiology of oblique clefts has commonly been explained by failed fusion of mesoderm in the embryonic facial processes; however, some of the lateral oro-ocular, some naso-ocular, and some of the medial oro-ocular clefts cannot be explained with the same. 10 Amniotic bands are implicated in etiology of these clefts. Rupture of amnion secondary to vascular insufficiency leads to formation of numerous fibrous bands which may interrupt the fusion of normal facial processes resulting in clefts. 6 13 14 The presentation of cleft palate in this case seems unique. The presence of a free lying uvula-like projection in the middle of the cleft with a connection to buccal mucosa over the alveolar ridge and absence of buccal sulcus in that area, with no connection to the nasal septum, could not be found anywhere else on extensive literature search using PubMed, Scopus, and other similar literature search tools. The cleft was bilateral and asymmetrical, and the lip was completely spared on the left side. Although the Tessier classification is most comprehensive, one must bear in mind that novel cleft combinations can occur.

## Conclusion

Treatment of rare craniofacial cleft is mostly individualized despite surgical algorithms. Surgical treatment of the defects should be done after proper assessment at an appropriate age to avoid functional and esthetic disturbances. Co-existing cleft lip or palate repair follows the same principles as in typical cases.
